# Long-term outcome after bilateral adrenalectomy in Cushing’s disease with focus on Nelson’s syndrome

**DOI:** 10.20945/2359-3997000000144

**Published:** 2019-06-19

**Authors:** Ana C. Cohen, Dolores Clifton Goldney, Karina Danilowicz, Marcos Manavela, María A. Rossi, Reynaldo M. Gómez, Graciela E. Cross, Oscar D. Bruno

**Affiliations:** 1 Universidad de Buenos Aires Division of Endocrinology Hospital de Clínicas University of Buenos Aires Buenos Aires Argentina Division of Endocrinology, Hospital de Clínicas, University of Buenos Aires, Buenos Aires, Argentina; 2 Foundation of Endocrinology Buenos Aires Argentina Foundation of Endocrinology (FUNDAENDO), Buenos Aires, Argentina

**Keywords:** Cushing’s disease, corticotrophinoma, Nelson’s syndrome, bilateral adrenalectomy

## Abstract

**Objective:**

We analyzed the clinical, biochemical, and imaging findings of adrenalectomized patients with Cushing’s disease (CD) in order to compare the characteristics of those who developed Nelson’s syndrome (NS) *versus* those who did not develop this complication (NNS), aiming to identify possible predictive factors for its occurrence.

**Subjects and methods:**

We performed a retrospective review of the clinical records of a group of patients with CD who underwent TBA between 1974 and 2011.

**Results:**

Out of 179 patients with CD, 13 (7.3%) underwent TBA. NS occurred in 6 of them (46%) after a mean of 24 months from the total bilateral adrenalectomy (TBA). Age at diagnosis, duration of Cushing’s syndrome (CS) until TBA, and steroid replacement doses were similar in both groups. Initial urinary cortisol levels (24-hour urinary free cortisol [UFC]) were significantly higher in the NS group than in the NNS group (*p* = 0.009). Four patients in the NS group and three of those in the NNS group received radiotherapy before TBA (*p* = 0.26). Three patients in the NS group presented residual tumors before TBA, compared with none in the NNS group (*p* = 0.04). At 1 year after TBA, the median ACTH level was 476 ng/L (240-1500 ng/L) in the NS group and 81 ng/L (48-330 ng/L) in the NNS group (*p* = 0.0007).

**Conclusion:**

In conclusion, a residual tumor before TBA, higher 24-hour UFC at diagnosis, and increasing ACTH levels within 1 year after TBA emerged as predictive factors of development of NS.

## INTRODUCTION

Cushing’s disease (CD) is caused by a corticotroph pituitary tumor. Patients with CD are usually treated with transsphenoidal surgery (TSS), as this approach leads to remission in 70-90% of the cases and is associated with low morbidity when performed by experienced pituitary surgeons (1).

However, the risk of CD recurrence 10 years after surgery can reach 20-25% of the patients in postoperative remission (2,3). Therefore, patients with persistent or recurrent CD might benefit from a second pituitary operation (which leads to remission in 50-70% of the cases), radiation therapy (RT) on the pituitary gland, or total bilateral adrenalectomy (TBA) (1,3). TBA offers an 85-100% success rate in controlling hypercortisolemia, and is particularly useful in patients with severe comorbidities associated with this condition (2,4,5). However, one of the limitations of TBA is the potential development of Nelson’s syndrome (NS), a possible life-threatening complication after TBA for refractory CD (1,2,4,6).

The diagnostic criteria of NS have varied widely since Don Nelson published his first series of patients in 1960 (7). Based on the available literature, NS is broadly defined as the association between pituitary tumor growth and progressive elevations of ACTH levels after bilateral adrenalectomy in patients with CD (8).

Many factors predicting the development of NS have been proposed, but the evidence is conflicting due to the heterogeneity of different reports.

The aim of this study was to compare the clinical, biochemical, and imaging features of patients who developed NS versus those who did not develop this complication to identify possible predictive factors for its occurrence.

## SUBJECTS AND METHODS

We performed a retrospective review of the clinical records of a group of patients with CD who underwent TBA between 1974 and 2011. In a cohort of 254 patients with Cushing’s syndrome (CS), 179 (70.4%) had an accurately confirmed diagnosis of CD, mostly by ACTH immunostaining of the tumor or inferior petrosal sinus sampling (IPSS). In those who did not undergo IPSS (particularly the early patients in the series), the diagnosis of CD was suggested by the metyrapone and 8-mg dexamethasone tests. Only 13 patients with CD (7.3%) were adrenalectomized and then followed up at the *Hospital de Clínicas* of the University of Buenos Aires. The mean follow-up after TBA was 14 years (range 5-30 years). Nine of the 13 patients (69%) were women and 4 (31%) were men, and the mean age at diagnosis of CD was 28 years (range 17-47 years). After surgery, all patients received standard doses of hydrocortisone and fludrocortisone.

We analyzed the patients’ clinical data, including age at TBA, gender, post-TBA hydrocortisone replacement doses, and previous pituitary irradiation. Biochemical features (24-hour urinary free cortisol [UFC] and ACTH levels before TBA, and ACTH levels after TBA), magnetic resonance imaging (MRI) before and after TBA, and complications during follow-up were also assessed. Patients were then classified into two groups according to the occurrence or absence of NS. This syndrome was defined according to the criteria proposed by Barber and cols. (5) as the presence of ACTH values >500 ng/L with rising levels on at least three consecutive occasions and/or an expanding pituitary mass lesion after TBA shown on MRI or CT scanning.

### Statistical analysis

The Wilcoxon rank sum test was used to compare the 24-hour UFC and ACTH levels, duration of CD prior to TBA, and steroid replacement doses in both groups. Fisher’s exact test was applied to assess the relationship between the development of NS and sex, age, presence of residual tumor, and RT prior to TBA. All statistical analyses were performed using Statistix 8.0 (Analytical Software, Tallahassee, FL, USA) and SPSS 16.0.0 (SPSS Inc., Chicago, IL, USA).

## RESULTS

In all, 13 patients underwent TBA between 1974 and 2011. TBA was performed by laparoscopy in 4 cases (31%) and by an open approach in 9 cases (69%). One patient undergoing laparoscopic surgery required conversion to open TBA. Immediate complications were hemorrhage in 1 patient (8%) and sepsis in 2 patients (15%), all of whom underwent adrenalectomy by an open approach. Long-term complications addressed were adrenal crisis in 2 (15%) of the patients and sepsis in 4 (31%) of them. NS occurred in 6 patients (46%) over a mean of 24 months (range 8-47 months) after TBA.

Clinical, biochemical, and imaging features of all patients are summarized in Table 1.

**Table 1:** Clinical, biochemical, and imaging features of the 13 adrenalectomized patients

Variable	Nelson’s syndrome	Non-Nelson’s syndrome	*p* value
Sex	Males: 2/6 Females: 4/6	Males: 2/7 Females: 4/7	1
Age at diagnosis of CD (years)	29.3 ± 11.8 (range: 18-47)	26.7 ± 6.9 (range: 17-37)	0.9
Mean follow-up after TBA (years)	13.1 ± 7.2 (range: 5-20)	26.7 ± 6.9 (range: 17-37)	1
Mean duration of CS prior to TBA (months)	21.5 ± 20.9 (range: 15-30)	37.1 ± 43 (range: 5-120)	0.73
Macroadenoma before TSS	Yes: 3/6	None: 0/7	
Persistent CD	Yes: 6/6	Yes: 6/7	1
Residual tumor before TBA	Yes: 3/6 (50%) No: 3/6 (50%)	Yes: 1/7 (14%) No: 6/7 (86%)	0.04*
RT prior to TBA	4/6 (66%)	3/7 (42%)	0.26
Steroid replacement dose (mg/dL)	20 ± 6 (range:15-30)	24 ± 5 (range: 20-30)	0.23
Mean initial 24-hour UFC (55 to 248 nmol/24h)	2291 (range: 1380-3533)	1139 (range: 287-3660)	0.009*
Mean initial ACTH levels (< 46 ng/L)	74 (range 33-133)	100.8 (range 12-305)	0.83
Median ACTH values 1 year after TBA (< 46 ng/L)	476 (range 240-1500)	81 (range:48-330)	0.0007*

CD: Cushing’s disease; TBA: total bilateral adrenalectomy; TSS: transsphenoidal surgery; RT: radiotherapy; UFC: urinary free cortisol; ACTH: adrenocorticotrophic hormone. Data are expressed as mean ± standard deviation or range values. * *p* values < 0.05 were considered significant.

NS was diagnosed in 6 patients (4 women and 2 men) with a mean age of 29.3 ± 11.8 years (range 18-47 years). In the 7 patients in the non-NS group (NNS) (5 women and 2 men) the mean age was 26.7 ± 6.9 years (range 17-37 years). There were no significant differences concerning age and sex distribution between both groups. The duration of CS prior to TBA was 21.5 ± 20.9 months in the NS group and 37.1 ± 43 months in the NNS group (*p* = 0.73).

**Figure 1:**
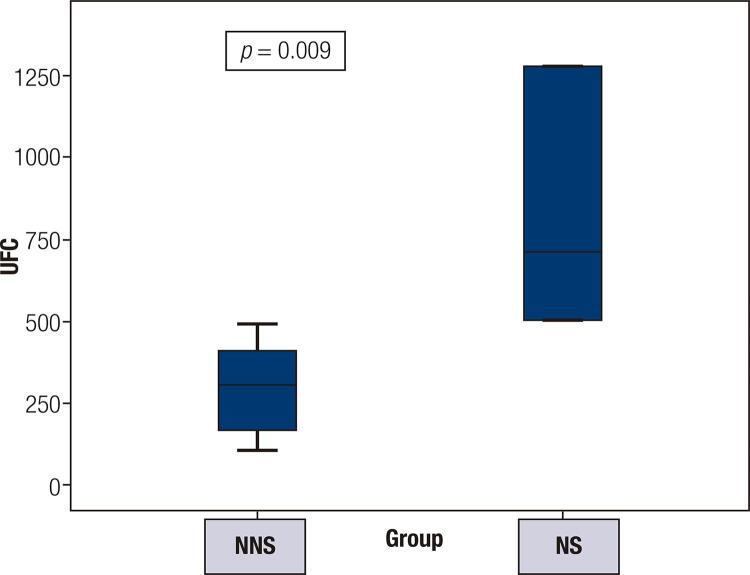
Initial 24-hour urinary free cortisol (UFC) levels.

NNS: non-Nelson’s syndrome; NS: Nelson’s syndrome. UFC levels are expressed in nmol/24h.

The 24-hour UFC levels at CD diagnosis were 2291 nmol/24h (range 1380-3533 nmol/24h) in the NS group and 1139 nmol/24h (range 287-3660 nmol/24h) in the NNS group (*p =* 0.009) (Figure 1). The mean ACTH levels at diagnosis were 74 ng/L (33-113 ng/L) in the NS group versus 100.8 ng/L (12-305 ng/L) in the NNS group (*p* = 0.83).

At the time of the diagnosis of CD, three out of six patients in the NS group harbored macroadenomas versus none in the NNS group. The remaining patients had microadenomas, except for one patient in the NNS group, who had no visible tumor on MRI, and one patient in the NS group treated early in the series, who had not undergone MRI imaging.

All patients in the NS group and 6 out of 7 of those in the NNS group had persistent, rather than recurrent CD (*p* = 1) before undergoing TBA.

Prior to TBA, RT was performed in 4 (67%) patients in the NS group and 3 (42%) of those in the NNS group (*p* = 0.26). In the former, the development of NS occurred after a mean of 54.7 months after RT (range 29-85 months). Two patients in the NS group received 20 Gy and 30 Gy of single-dose stereotaxic radiosurgery and fractionated conventional RT (FCRT), respectively. One patient in the NNS group received 50 Gy of FCRT. There were no data related to the RT doses in the remaining five patients in this series.

Three (50%) patients in the NS group and one (14%) patient in the NNS group presented residual tumors before TBA (*p* = 0.04).

The doses of hydrocortisone replacement administered after TBA were 20 ± 6 mg/day and 24 ± 5 mg/day in the NS and NNS groups, respectively (*p* = 0.23).

**Figure 2:**
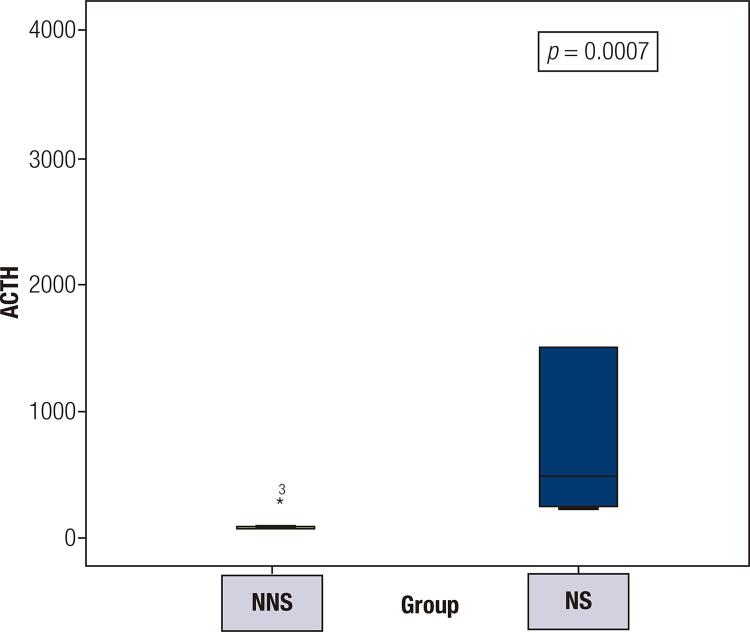
Adrenocorticotrophic hormone (ACTH) levels 1 year after total bilateral adrenalectomy (TBA).

NNS: non-Nelson’s syndrome; NS: Nelson’s syndrome. ACTH levels are expressed in ng/L.

At 1 year after TBA, the median ACTH values were 476 ng/L (range 240-1500 ng/L) in the NS group and 81 ng/L in the NNS group (range 48-330 ng/L; *p* = 0.0007) (Figure 2).

**Figure 3:**
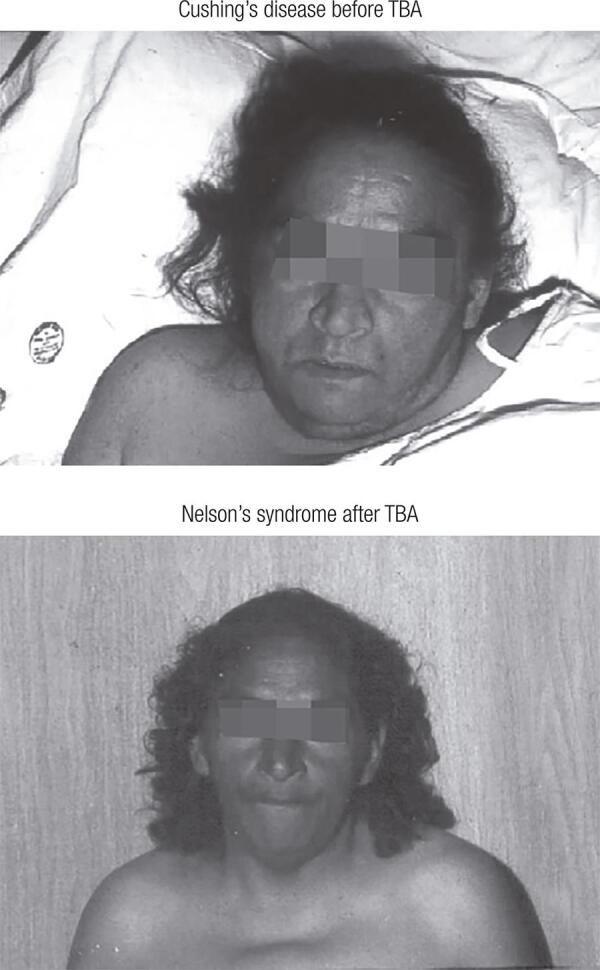
Female patient showing intense hyperpigmentation after total bilateral adrenalectomy (TBA) due to Cushing’s disease.

During follow-up, all patients with NS developed hyperpigmentation, including one patient who presented an ACTH value of 6800 ng/L (Figure 3), and three patients who experienced tumor progression. One patient, with a survival time of 2 years, died because of tumor complications of NS after undergoing emergency surgery due to tumor bleeding and several pharmacological treatments. Only two patients with NS achieved remission: one after transcranial surgery and the other after conventional radiotherapy. One patient was diagnosed only recently and had not received any treatment until the publication of this article. The other patients received different treatment modalities and had no tumor progression up to this publication. Table 2 summarizes the follow-up data of all patients.

**Table 2:** Follow-up data of patients with Nelson’s syndrome

Patient	Previous treatments for CD	Follow-up since NS diagnosis	NS diagnostic criteria	Medical treatments for NS	Surgical treatments for NS	RT treatment	Outcomes
1	1 TSS 1 TCS SRS (20 Gy)	4 years	ACTH increase	CBG 4 mg (1 year)	No	No	Initial response, reduced ACTH values. In 2015: ACTH > 1250 ng/L, no tumor found on MRI
2	1 TSS RT^(1)^	20 years	Tumor growth and ACTH increase	No	TCS	No	Remission
3	1 TCS	10 years	ACTH increase	No	No	RT with gold implant (1977)	Remission
4	1 TSS RT^(1)^	4 months	ACTH increase	No	No	No	No treatment yet. Recently diagnosed.
5	1 TSS Conventional RT (30 Gy)	3 years, 6 months	Tumor growth and ACTH increase	CBG 2 mg (5 months) Octreotide 20 µg (3 months) Pasireotide 60 mg (1 month)	Emergency TSS for tumor bleeding (Ki67 30%)	No	Deceased due to complications related to the tumor.
6	2 TSS	16 years	Tumor growth and ACTH increase	CBG intolerance TMZ (6 cycles of 1250 mg) Octreotide 20 mg (10 months)	No	GKRS (70 Gy)	Tumor stability. Asymptomatic.

CD: Cushing’s disease; ACTH: adrenocorticotrophic hormone; TSS: transsphenoidal surgery; TCS: transcranial surgery; TBA: total bilateral adrenalectomy; CBG: cabergoline; TMZ: temozolamide; SRS: stereotaxic radiosurgery; RT: radiotherapy; GKRS: gamma knife radiosurgery. ^(1)^ No data available on the radiotherapeutic doses.

## DISCUSSION

Bilateral adrenalectomy may be an alternative treatment for patients with refractory CD. This is an effective, potentially lifesaving option for controlling hypercortisolism in almost all patients. In a German series, the frequency of TBA was reported at 18% among 124 patients with CD, while in an Italian multicenter study, the frequency reached 10% of 288 cases of CD (6,^9^). Similar to the latter study, 7.3% of the patients with CD in our series underwent TBA. Regarding the estimated risk of postoperative complications after TBA, a recent systematic review including 23 studies featuring 739 patients with CS estimated the risk to be about 18% (6). Laparoscopic adrenalectomy is associated with a shorter hospital stay and a reduced period of rehabilitation compared with open surgery (10). In our series, open TBA was performed in most patients because the laparoscopic approach was not available at our hospital until the late 1990s. Aligned with the literature on this topic, only 23% of our patients had immediate complications, all of whom occurred after open TBA.

In previous reports, the prevalence of NS varied between 8-29% in the largest series (11-14), with an interval of 0.5-24 years between the TBA and NS diagnosis (15). However, Assié and cols. (16) reported a prevalence of NS of 47% in a series of 53 adrenalectomized patients with CD and, more recently, Graffeo and cols. reported the prevalence to be 53% (17). This higher occurrence may be explained by the fact that NS was diagnosed based on the concept of corticotroph tumor progression rather than the presence of an extensive sellar mass alone.

Most patients developed NS during the first years after TBA, as described by several authors (5,11,15). Gil-Cárdenas and cols. (15) reported the occurrence of NS after a mean of 15 months (range 2-33 months) after surgery, while in a series by Kelly and cols. (18), this complication occurred after a mean of 5 years (range 1-25 years). Our series reported an incidence of NS of 46% after a mean of 22 months (range 8-40 months) after TBA based on the two main criteria proposed by Barber and cols. (5), namely, ACTH values > 500 ng/L with progressive elevation (an ACTH increase > 30% of the initial result after TBA) on at least three consecutive occasions and/or an expanding pituitary lesion after TBA. Concerning the definition of NS, although more than 100 cases have been documented in reports and short series, definitive diagnostic criteria for NS have not been established yet (17). The diagnosis of NS is controversial, but the existing criteria focus on an enlarging pituitary tumor after TBA (the appearance of a new adenoma or progression of the remnant corticotroph) and increasing ACTH levels (5,17). Addressing the former issue, Assié and cols. defined corticotroph tumor progression as a growth of at least 2 mm in one of the three dimensions of a remnant adenoma (16). However, these criteria can only be applied to good quality MRI scans read by experienced radiologists, which could not be done in some of the patients, taking into account the retrospective nature of our study. On the other hand, the ACTH criteria have been supported by several series (11,14,19). The addition of the criterion of 30% increase prevents a diagnosis of NS from being established on the basis of relatively small and clinically insignificant progressive increases in ACTH that may occur by chance or assay variability (5).

Much effort has been directed towards the identification of predictive factors for the development of NS. Different authors have reported no correlation between gender and NS occurrence (18-20). Aligned with that, we found no differences in gender between the NS and NNS groups in our series.

A younger age at the moment of the TBA did not seem to be a predictive factor for the development of NS in the present study. Previous reports are conflicting in this sense. A group of authors has hypothesized that since aggressive corticotrophinomas are more likely to occur in children, NS may develop more frequently in patients who are adrenalectomized at a younger age (21). Following this theory, Kasperlik-Załuska and cols. (14) and Kemink and cols. (22) suggested the age of 30 and 35 years**,** respectively, as cutoff ages for an increased risk of NS development. Graffeo and cols. also identified age as a risk factor for NS (17), but several authors (11,16,19) were unable to find a significant difference between younger age and the development of NS. Therefore, new and larger series are needed to confirm these findings.

The duration of CD before TBA and its relation to the occurrence of NS is another controversial point. In one study of 43 patients, NS developed in those who had had symptoms of CD for a longer time prior to TBA (23), while in another series of seven patients, no association was observed between the duration of CD before TBA and the occurrence of NS (24). Conversely, other authors (16,25) have found that a shorter interval between the onset of CD and TBA was related to an increased incidence of NS. One explanation for this fact may be that patients with more aggressive tumors require earlier TBA. In this regard, no significant differences were observed between our groups.

Higher 24-hour UFC levels before TBA were predictive of NS in our series. Some authors defend the pre-TBA UFC level to be a useful marker of tumor size and functionality and a predictor of subsequent NS development (11,26). However, this evidence is not conclusive since such correlation has not been demonstrated in some studies (19,20,23).

Concerning ACTH levels prior to TBA, we found no significant differences between both groups. These findings are in line with those of a recent series of 88 adrenalectomized patients published by Graffeo and cols. (17).

Regarding steroid replacement doses, some authors postulate that insufficient doses after TBA may favor the occurrence of NS due to lack of a negative feedback from the pituitary-adrenal axis (11,14,27). On the other hand, Kelly and cols. (23) and Barnett and cols. (20) reported no relationship between insufficient glucocorticoid doses and the risk of NS development. In agreement with the latter, we found no significant association between an insufficient glucocorticoid dose and the development of NS.

As a strategy for preventing or delaying NS development, RT is not universally accepted. Gil-Cárdenas and cols. (15) described a series of 39 adrenalectomized patients followed up for 15 years, in which 50% who did not receive RT developed NS compared with none of the patients who did so. Two other series consisting of 20 and 75 adrenalectomized patients have supported the protective role of RT (13,28). Nagesser and cols. (11) concluded that the administration of RT could delay the occurrence of NS (*p* = 0.025). In one of the latest publications, Mehta and cols. (29) reported a series that included 20 adrenalectomized patients with persistent CD after transsphenoidal surgery and gamma knife radiosurgery (GKRS). The patients were followed up with MRI for a median of 5.4 years. Only one patient (5%) demonstrated tumor growth, defined by a 10% increase in the original tumor volume, which occurred 9 months after TBA. They concluded that the use of GKRS before TBA reduced the incidence of NS (5.3% at 3 years and 7 years) by using MRI sequences to detect tumor growth in comparison with historical controls (39% and 47% at 3 and 7 years, respectively) (16).

In contrast, Dornhorst and cols. (30) described a series of 38 patients who underwent TBA due to CD, 20 of whom were irradiated. Overall, 29% of the irradiated patients and 50% of the non-irradiated ones developed NS, with no significant difference between both groups. Recently, Graffeo and cols. found in a group of 88 adrenalectomized patients that a history of RT prior to TBA was predictive of NS (17). However, this was only evidenced in patients who experienced tumor recurrence before TBA, whereas in patients who had tumor persistence, no significant differences were observed among those who received RT versus those who did not. This observation is consistent with our findings, since most of our patients (12 out of 13) had tumor persistence.

Finally, Pereira and cols. (19) found no benefit of prophylactic RT, although some of their patients did not receive the conventional RT doses used in patients with CD (50 Gy) or the RT was not administered in the correct way or amount. Similarly, in our series, two of four patients who developed NS and were irradiated before TBA received insufficient RT doses, while no data were available on the doses received by the other two patients.

In this series, the administration of RT prior to TBA appeared to have no relationship with the development of NS but, since complete data are lacking, we failed to arrive at well-validated conclusions.

In relation to ACTH levels 1 year after TBA, we found a significant connection between a rapid increase in ACTH levels and the incidence of NS. Currently, in most studies, this is probably the best-validated predictive factor for the occurrence of NS, and may be related to tumor progression (15,16,19,20). Concerning the presence of residual tumor before TBA, Pereira and cols. (19) concluded that adrenalectomized patients who presented evidence of tumor remnant before TBA developed NS more frequently than did those without remnants (30% *versus* 17%, respectively), even though no significant difference was found in this regard. Sonino and cols. (26) showed that 41.6% of the patients with residual tumor prior to TBA were diagnosed with NS compared with none without remnants (*p* < 0.01). Therefore, the presence of residual tumor before TBA could be considered a potential predictive factor for the occurrence of NS. Aligned with that, we found a significant association between the presence of residual tumor and the development of NS.

Our study has several weaknesses. Firstly, due to its retrospective design, we had to rely on clinical records that in some cases were incomplete. Some specific immunohistochemistry data, such as the Ki67 index, was not available in most samples, nor were the RT doses used in two cases. Because of the long period of patient recruitment (1974-2011), only one patient (in the NS group) was not initially evaluated by MRI or CT scanning. Nevertheless, the patient had fulfilled the ACTH criterion to define NS.

Although the number of patients with TBA in this series is rather small, it reflects the actual number of cases of CD (n = 161) followed up in an Argentinian reference hospital that fulfills the criteria of a Pituitary Tumor Center of Excellence (31). The strength of this study is that it provides a unique, single-center, long-term experience in the follow-up of adrenalectomized patients in Latin America.

In conclusion, bilateral adrenalectomy is a well-known therapeutic strategy for patients with refractory CD. Although it can provide an immediate control of hypercortisolism and is a relatively safe procedure, it can lead to severe, life-threatening side effects like NS.

The presence of tumor remnants before adrenal surgery and the increase in ACTH levels in the first year after TBA are two predictive factors that should be considered in these patients. As the emergence of NS usually occurs in the first 3 years, ACTH level monitoring and periodic MRI scanning should be performed more frequently for earlier detection of this complication. However, taking into account that the occurrence of NS has been described up to 24 years after TBA, lifelong surveillance may be justified.

Finally, we found that higher 24-hour UFC levels before TBA could be a predictive factor of NS occurrence. Therefore, it should be evaluated and prioritized in patients considered for TBA.

Our findings highlight the importance of the selection of CD patients for TBA, taking into consideration the presence of potential predictive factors for NS in each patient.
